# Abyssinone V-4′ Methyl Ether, a Flavanone Isolated from *Erythrina droogmansiana*, Exhibits Cytotoxic Effects on Human Breast Cancer Cells by Induction of Apoptosis and Suppression of Invasion

**DOI:** 10.1155/2020/6454853

**Published:** 2020-07-23

**Authors:** Stéphane Zingue, Abel Joël Gbaweng Yaya, Julia Cisilotto, Larissa Vanelle Kenmogne, Emmanuel Talla, Anupam Bishayee, Dieudonne Njamen, Tânia Beatriz Creczynski-Pasa, Derek Tantoh Ndinteh

**Affiliations:** ^1^Department of Life and Earth Sciences, Higher Teachers' Training College, University of Maroua, P.O. Box 55, Maroua, Cameroon; ^2^Department of Pharmaceutical Sciences, Health Sciences Centre, Federal University of Santa Catarina, Florianópolis, Santa Catarina, Brazil; ^3^Department of Chemical Sciences, Faculty of Science, University of Johannesburg, Doornfontein 2028, South Africa; ^4^Department of Chemistry, Faculty of Science, University of Ngaoundere, P.O. Box 454, Ngaoundere, Cameroon; ^5^Department of Animal Biology and Physiology, Faculty of Science, University of Yaoundé 1, P.O. Box 812, Yaoundé, Cameroon; ^6^Lake Erie College of Osteopathic Medicine, Bradenton, FL 34211, USA

## Abstract

Abyssinone V-4′ methyl ether (AVME) isolated from *Erythrina droogmansiana* was recently reported to exhibit anti-mammary tumor effect in mice. The present work was therefore aimed at elucidating its cellular and molecular mechanisms. To achieve our goal, the cytotoxicity of AVME against tumoral and non-tumoral cell lines was evaluated by resazurin reduction test; flow cytometry allowed us to evaluate the cell cycle and mechanisms of cell death; the mitochondrial transmembrane potential, reactive oxygen species (ROS) levels, and caspase activities as well as apoptosis-regulatory proteins (Bcl-2 and Bcl-XL) were measured in MDA-MB-231 cells. Further, the antimetastatic potential of AVME was evaluated by invasion assay. AVME exhibited cytotoxic effects in all tested tumor cell lines and induced a significant increase in the percentage of MDA-MB-231 cells at G2/M and S phases of the cell cycle in a concentration-dependent manner. AVME also induced apoptosis in MDA-MB-231 cells, which was accompanied by the activation of caspase-3 and caspase-9 and downregulation of Bcl-2 and Bcl-XL proteins. Moreover, AVME suppressed cancer cell invasion by the inhibition of the metalloproteinase-9 activity. Findings from this study suggest that AVME has anti-breast cancer activities expressed through mitochondrial proapoptotic pathway including impairment of aggressive behaviors of breast cancer cells.

## 1. Introduction

The most common cancer in women is breast cancer which represents 29% of all diagnosed cancers in women [1]. Global estimates indicate that one million women are diagnosed with breast cancer each year and more than 400,000 of them die of this disease [2]. In Cameroon, 2,625 new cases of breast cancer are diagnosed in women each year [3,4]. Despite considerable advancement in medical care, deaths resulting from breast cancer are still on the increase [5]. This is particularly the situation in developing countries where governments are less ready to face this threat as a result of scarcity of diagnostic tools and the high cost of treatments [6]. Nevertheless, in first world countries, the problem of resistance and high cytotoxicity of many conventional drugs is one of the greatest difficulties that anticancer therapies are facing [7]. Therefore, majority of cancer patients usually incorporate natural therapy into conventional treatment protocols [8]. However, due to the lack of scientific evidence, the benefit of such substances is yet to be established. This is particularly true of phytoestrogens which are plant metabolites with a chemical structure of 17*β*-estradiol, which mimic estrogenic actions in mammals [9]. Since phytoestrogens possess both estrogenic and antiestrogenic activities, it is proposed that they could prevent estrogen-dependent malignancies such as breast, ovarian, uterine, and prostate cancers [10].

*Erythrina* (Fabaceae) contains more than 100 species distributed in the tropics and subtropics of America, Africa, and Australasia [11]. Extracts from *Erythrina* spp. exhibit a wide range of pharmacological properties, including cytotoxic [12, 13] and phytoestrogenic activities [14–17]. Among the most abundant metabolites isolated from this genus are abyssinones, which are prenylated flavanones that possess aromatase-inhibitory (abyssinone II), antioxidant and cytotoxic (abyssinone I and II), and anti-inflammatory (abyssinone V-4′ methyl ether) activities [18–21]. Abyssinone V-4′ methyl ether (AVME, Table 1) also possesses estrogenic and antiestrogenic effects [15, 22]. Recently, Tueche et al. [23] reported the cytotoxic effect of AVME isolated from *Erythrina droogmansiana* on four tumoral cell lines [including estrogen receptor-positive breast adenocarcinoma (MCF-7)] and its ability to prevent breast tumors induced by 7,12-dimethylbenz(a)anthracene (DMBA) in mice. Given its aforementioned antiestrogenic and cytotoxic effects, AVME might be a good candidate for the treatment of estrogen-dependent cancers, mainly breast cancer. As the information available on the cellular and molecular mechanisms of AVME on cancer cells is limited, this study aimed to better understand the underlying mechanisms of the anticancer activity of AVME. To achieve our goal, cell death (apoptosis or necrosis), cell cycle, mitochondrial transmembrane potential, ROS formation, caspase activities, apoptotic regulating proteins (Bcl-2 and Bcl-XL), invasion and expression of its regulators, matrix metalloproteinase-2 (MMP-2), and MMP-9 were examined in MDA-MB-231 breast cancer cells.

## 2. Materials and Methods

### 2.1. Chemicals and Reagents

The following reagents were obtained from Sigma-Aldrich (St. Louis, MO, USA): acridine orange, trypan blue, resazurin, ethidium bromide, and cell culture mediums. Fetal bovine serum (FBS) and antibiotics were obtained from GIBCO (Grand Island, NY, USA). ApopNexin™ FITC Apoptosis Detection Kit was obtained from Millipore (Billerica, MA, USA). 2-[4-(2-Hydroxyethyl)piperazin-1-yl]ethanesulfonic acid (HEPES) was obtained from Ludwig Biotecnologia Ltda. (Alvorada, RS, Brazil). Millicell® cell culture inserts (8.0 *µ*m) were obtained from Merck Millipore LTD (Tullagreen, Carrigtwohill, Ireland). JC-1 (5,5′,6′,6-Tetrachloro-1,1′, 3,3′-tetraethylbenzymidazolcarbocianyne iodide) and DCFH-DA were obtained from Invitrogen (Carlsbad, CA, USA). The antibodies Bcl-2 (monoclonal, C2; sc-7382), Bcl-XL (monoclonal, H5; sc-8392), and *β*-actin (monoclonal, C4; sc-47778) were obtained from Santa Cruz Biotechnology, Inc. (Santa Cruz, CA, USA). All solutions were prepared using ultrapure water.

### 2.2. Collection and Authentication of Plant Material

*Erythrina droogmansiana* T. Durand (Fabaceae) root bark was harvested from Nkomekoui, Yaoundé, Centre Region of Cameroon, on August 21, 2010 (∼8:00 a.m.). It was identified by Mr. Victor Nana, a botanist in the Cameroon National Herbarium where a voucher specimen (no. 4261/SRFK) was preserved.

### 2.3. Extract Preparation

The root bark of *E*. *droogmansiana* was air-dried and macerated to produce a powder. Then, 1.2 kg of the powdered material was added with 5 L of ethyl acetate and incubated for 48 h at room temperature for extraction purposes. The mixture was filtered through Whatman filter paper no. 4. Ethyl acetate was recovered using a rotary evaporator, and 150 g (12.5%) of crude extract was obtained.

### 2.4. Isolation of AVME

The isolation of AVME has been previously reported by Tueche et al. [23]. Briefly, 100 g of the ethyl acetate extract was subjected to column chromatography over silica gel packed in n-hexane. Gradient elution was carried out in increasing polarity using n-hexane, ethyl acetate, and methanol to obtain seven series of fractions that were mixed based on their respective thin layer chromatographic (TLC) profiles. Column elution with the solvent system hexane-EtOAc (90:10) yielded YG4 and other compounds. Chemical structures were elucidated by spectral methods (MS, NMR, and element analysis). Compound YG_4_ was a white powder (500 mg), with an [M]^+^ at *m*/*z* 422.2094 corresponding to the molecular formula C_26_H_30_O_5_. This compound was identified as AVME (Table 1). The presence of a flavanone skeleton was evident from the ^1^HNMR spectra at *δ* 5.27 (1H, br) and 2.68 (1H, dd, *J* = 2.8, and 17.2 Hz) and at *δ* 3.06 (1H, dd, *J* = 13.2 and 17.2), corresponding to H-2 and H-3 protons of the C-ring of the flavanones, respectively. From the ^13^CNMR spectra, the presence of a signal at 79.3 and 42.5 indicated the C-2 and C-3 of the C-ring of flavanones, respectively. The ^1^H and ^13^CNMR spectra data of AVME were compared with spectra already shown elsewhere [24].

### 2.5. Culture of Cell Lines

The following cell lines were acquired from the Rio de Janeiro Cell Bank (Federal University of Rio de Janeiro, Rio de Janeiro, Brazil): MCF-7 [human estrogen receptor- (ER-) positive breast carcinoma cells], MDA-MB-231 (human ER-negative breast carcinoma cells), 4T1 (mouse mammary tumor cells), SK-MEL-28 (human melanoma cells), SF-295 (human glioblastoma cells), HUVEC (human umbilical vein endothelium cells), MCR-5 (human fetal lung fibroblast cells), and NIH/3T3 (murine fibroblast cells).

MDA-MB-231, MCR-5, and SK-MEL-28 cell lines were cultured in Dulbecco's modified Eagle's medium (DMEM) containing 10% FBS. RPMI 1640 medium with 10% FBS was used for the growth and subculture of MCF-7, 4T1, HUVEC, NIH/3T3, and SF-295 cells. Each culture medium was supplemented with 100 U/mL penicillin, 100 *µ*g/mL streptomycin, and 10 mM HEPES. All cells were maintained at 37°C in a 5% CO_2_ humidified atmosphere and pH 7.4. Every two days, cells were passaged. The number of viable cells was estimated at the beginning of each experiment by trypan blue method using a Neubauer chamber.

### 2.6. Resazurin Reduction Assay

The cytotoxic effect of AVME was investigated by Alamar Blue (resazurin salt) assay following the method of O'Brien et al. [25]. Briefly, 1 × 10^4^ cells in culture medium were seeded in each well onto a 96-well plate overnight. AVME (5 to 100 *µ*M) was then added to the seeded cells on subconfluent cell culture and incubated for 24 h. Fluorescent intensity was captured using Perkin Elmer LS55 spectrofluorimeter (Becton Dickinson, San Jose, CA, USA) with excitation/emission wavelengths of 530/590 nm. The CC_50_ (cytotoxic concentration that kills 50% of the cells) was calculated by nonlinear regression analysis of the logarithm of concentration with respect to the normalized response using SigmaPlot 11.0 software. Each experiment was done in triplicate and repeated thrice. The results were expressed as a percentage of cell viability. The effect of AVME in tumoral and nontumoral cells was compared using the selective cytotoxicity index (SCI), which was determined as described by Robles-Escajeda et al. [26] using the following formula: SCI = CC_50_ of nontumoral cells/CC_50_ of tumoral cells. For this calculation, the HUVEC cells were used as a representative of nontumoral cells.

### 2.7. Morphological Identification for Cell Death

Qualitative characterization of cell death mechanism was done using ethidium bromide (EB, 3,8-diamino-5-ethyl-6-phenylphenanthridinium bromide) and acridine orange (AO, 3,6-dimethylamino acridine) stains. Cells containing intact membranes fixed AO in their DNA and emitted green fluorescence, while cells with damaged membranes emitted red fluorescence. MDA-MB-231 cells were seeded onto 12-well plates (3.5 × 10^5^ cells/mL) on subconfluent cell culture and incubated with AVME at 10 or 20 *µ*M or DMSO (solvent control) for 24 h. After incubation, the medium was decanted and the cells were washed with phosphate-buffered saline (PBS). Subsequently, the mixed dye (0.3 *µ*g/mL of AO and 1 *µ*g/mL of EB) was added to each well, and the cells were viewed immediately under a Nikon eclipse TS100 inverted microscope at 100 and 400× magnifications with excitation filter 480/30 nm, dichromatic mirror cut-on 505 nm LP, and barrier filter 535/40 nm. Images were captured using a Nikon COOLPIX digital camera connected to a computer and analyzed by image editor software (ImageJ®).

### 2.8. Flow Cytometry for Detection of Apoptotic Cells

Annexin FITC-conjugated (1 : 500) and propidium iodide (PI) fluorochrome-labeled cells were used to determine the nature of cell death (apoptosis or necrosis). After platting MCF-7 and MDA-MB-231 cells (3.5 × 10^5^ cells/mL) in a 12-well plates, they were incubated with AVME at 10 and 20 *µ*M for MDA-MB-231 cells and 11 and 22 *µ*M for MCF-7, or solvent control (DMSO) for 24 h. Cells were then washed twice with cold PBS and suspended again in a buffer containing 10 mM HEPES (pH 7.4), 150 mM NaCl, 5 mM KCl, 1 mM MgCl_2_, and 1.8 mM CaCl_2_. The pellets were stained on ice in the dark for 15 min with a fluorescent probe solution containing 50 mg/mL PI and 1 mg/mL Annexin. The total percentage of cells experiencing apoptosis was demarcated as the sum of both early and late facets of apoptosis (Annexin V-FITC positive), lower and upper right quadrants in the two-parameter dot plots, as previously described by Robles-Escajeda et al. [26]. The WinMDI 2.9 software and the flow cytometer BD FACSVerse (Becton Dickinson, Franklin Lakes, NJ, USA) were used for analysis. Each experiment was repeated thrice.

### 2.9. Cell Cycle Analysis

MCF-7 and MDA-MB-231 cells were plated onto 12-well plates (3.5 × 10^5^ cells/mL) and incubated for 24 h. After replacing the medium, AVME was added at concentrations of 10 and 20 *µ*M (for MDA-MB-231) or 11 and 22 *µ*M (for MCF-7) or solvent control (DMSO) and incubated for 24 h. The cells were washed several times with cold PBS, resuspended in 70% ethanol, and fixed at −20°C. A mixture of PBS and 2% bovine serum albumin (BSA) was added after 30 min. Cell pellets obtained after centrifugation were washed, permeabilized with a lysis buffer (0.1% Triton X-100 and 100 *µ*g/mL RNase), and stained with PI (20 *µ*g/mL). Cell cycle was assayed using a flow cytometry (BD FACSVerse, Becton Dickinson, Franklin Lakes, NJ, USA), and results were analyzed using WinMDI 2.9 software. Each experiment was repeated thrice.

### 2.10. Analysis of Mitochondrial Membrane Potential (ΔΨm)

The probe JC-1 (5,5′,6′6-tetrachloro-1,1′,3,3′-tetraethylbenzymidazolcarbocianine iodide), a lipophilic cationic fluorochrome, was used to measure the mitochondrial transmembrane potential of MDA-MB-231 cells. MDA-MB-231 cells were seeded onto 12-well plates (3.5 × 10^5^ cells/mL) and incubated for 24 h. Thereafter, AVME (at 10 and 20 *µ*M), DMSO (solvent control), or uncoupler CCCP (positive control) was added to cells on subconfluent cell culture. After 6 h of incubation, JC-1 (10 *μ*g/mL) was added and cells were further incubated for 30 min at 37°C in 5% CO_2_. The cells were then washed twice with PBS and resuspended in PBS. A Perkin Elmer LS55 spectrofluorimeter (Becton Dickinson, San Jose, CA) was used to measure the fluorescence intensities. The excitation/emission wavelength for JC-1 was 488/525 nm. The mitochondrial potential was calculated by the ratio of 590/525 fluorescence intensities and compared with the control cells with 100% ΔΨm. Each experiment was performed thrice.

### 2.11. Measurement of ROS

2',7′-Dichlorodihydrofluorescein diacetate (DCHF-DA) was used to evaluate the formation of intracellular free radicals. DCHF-DA is oxidized to dichlorofluorescein (DCF) in the presence of ROS. Briefly, MDA-MB-231 cells (3.5 × 10^5^ cells/mL) were seeded onto 12-well plates and incubated for 24 h. Thereafter, the medium was decanted, and AVME at 10 and 20 *µ*M or solvent control (DMSO) was added to the wells on subconfluent cell culture. After 12 h incubation, cells were treated with a medium containing 10 *μ*M DCHF-DA. The cells were then washed four times with cold PBS, detached by trypsinization, and centrifuged at 600 × g for 10 min. PBS-EDTA was used to suspend cell pellets whose DCF fluorescence signal was detected using a spectrofluorimeter (Perkin Elmer LS55, Becton Dickinson, San Jose, CA, USA) with excitation at 485 nm and emission at 535 nm. Fluorescence units were expressed as the percentage of ROS and normalized by the total protein content measured by a modified method described by Lowry et al. [27].

### 2.12. Determination of Caspase Activities

Caspase-3, caspase-9, and caspase-8 activities were measured after incubation of 2 × 10^6^ MDA-MB-231 cells for 8 h at 37°C with AVME at concentrations of 10 and 20 *µ*M. Cells were washed twice with PBS and then subjected to lysis in a buffer prepared as follows: 1 *µ*g/mL pepstatin A, 5 *µ*g/mL aprotinin, 5 mM 3-[(3-cholamidopropyl)-dimethylammonio]-1-propanesulfonate (CHAPS), 50 mM HEPES (pH 7.4), 5 mM dithiothreitol (DTT), 1 mM phenylmethylsulfonyl fluoride (PMSF), and 1 *µ*g/mL leupeptin at 8°C for 20 min. An aliquot of AVME added to a buffer containing 20 mM HEPES (pH 7.4), 0.1% CHAPS, 2 mM EDTA, 5 mM DTT, and 5% sucrose was added to the cells. The reaction medium was complemented with 50 *µ*M Ac-DEVD-AMC, 100 *µ*M Ac-LEHD-AFC, or 25 *µ*M of Ac-IETD-AMC, fluorogenic substrates for caspase-3, caspase-9, and caspase-8, respectively. The caspase activities were therefore determined spectrofluorimetrically by the production of 7-amino-4-methyl coumarin (AMC) or 7-amino-4-trifluoromethylcoumarin (AFC) after 2 h incubation at 37°C. The protein content was assayed as indicated earlier. The caspase activities were expressed as percentages based on the values of fluorescent units normalized with the protein concentration.

### 2.13. Western Blot Analysis

MDA-MB-231 cells were seeded in a density of 1 × 10^7^ cells/well onto a 6-well plate. After complete cell attachment within 24 h, AVME was added at concentrations of 10 and 20 *µ*M on subconﬂuent cell culture and incubated for 24 h. The cells were then broken in a lysis buffer, sonicated, and centrifuged for 10 min at 10,000 × g to extract the proteins. The protein content was determined as mentioned previously. Sodium dodecyl sulfate-polyacrylamide gel electrophoresis (SDS-PAGE) using a 15% gel was performed by loading aliquots of 50 *µ*g of protein from each sample. The separated protein bands were transferred to nitrocellulose membranes which were blocked with 5% BSA in Tris-buffered saline containing Tween 20 (TBS-T). The membranes were incubated overnight at 4°C with primary-human specific monoclonal antibodies for Bcl-2, Bcl-XL, or *β*-actin (at a 1 : 1,000 dilution) in TBS-T containing 2.5% BSA. Subsequently, the membranes were washed four times with TBS-T and incubated with the anti-mouse IgG-peroxidase conjugated secondary antibody (1 : 10,000 dilution). Immune complexes were observed by chemiluminescence using Amersham ECL™ (GE Healthcare, London, UK), Western blotting detection reagent, and ChemiDoc MP imaging system (Bio-Rad, St. Louis, MO, USA). Proteins were quantified using Bio-Rad image analysis software. In this experiment, *β*-actin served as internal control.

### 2.14. Cell Invasion Assay

Matrigel precoated Millicell culture inserts (8.0 *µ*m pores, 10 mm diameter) contained in 24-well plates were used to assess chemotaxis. Before the experiment, inserts were washed twice with DMEM and rehydrated for 30 min in DMEM. An aliquot of Matrigel (1 : 10 in DMEM without serum) was added to the inserts. The insert with Matrigel was incubated at 37°C for 1 h to enable polymerization. A suspension of 5 × 10^4^ MDA-MB-231 cells was homogenously added in the upper chamber whereas the lower chamber was filled with DMEM containing 10% FBS. Cells were incubated for 48 h in the presence of AVME (10 and 20 *μ*M) or DMSO (control) to allow them to migrate. Afterward, the upper surface of the transwell membrane was wiped gently with a cotton swab to remove nonmigrating cells and fixed with 5% glutaraldehyde followed by staining with 0.1% crystal violet solution for 10 min. Images were captured with a Nikon COOLPIX digital camera. A minimum of five fields per insert was counted by image editor software (ImageJ®). Each experiment was performed twice, and the average of cells/field was calculated. The number of untreated cells that invaded was considered to be 100%, and the percentage of cells treated with AVME was calculated based on the number of cells in the control group.

### 2.15. Determination of Metalloproteinase Activities

A zymography assay was performed to determine the activity of MMP-2 and MMP-9 as reported by Cisilotto et al. [28]. Briefly, 3 × 10^5^ cells/well were seeded, and, after incubation with AVME at 10 and 20 *µ*M for 24 h in serum-free medium, the culture medium containing the metalloproteinases released by the cells was removed. Samples were centrifuged at 8000 × g for 20 min at 4°C to remove dead cells and cell fragments. Total protein was measured by a modified method described by Lowry et al. [27]. The proteins (30 *µ*g) were separated by electrophoresis using 10% polyacrylamide gel with 0.5% of gelatin. Thereafter, gels were treated with 2.5% Triton X-100 with gentle agitation for 30 min at room temperature. Gels were further incubated in a buffer with 0.05 M Tris-HCl, 10 mM CaCl_2_, and 1 *µ*M ZnCl_2_ (pH 8.0) overnight at 37°C. Gels were stained with a Coomassie solution (0.5% Coomassie Blue, 30% methanol, and 10% acetic acid) for 30 min and eventually destained with a solution containing 10% methanol and 10% acetic acid. Zymographic images were processed using ENDURO™ GDS photodocumentation system (Labnet International, Edison, NJ, USA). Protein bands were quantified using the ImageJ® software.

### 2.16. Statistical Analysis

Results are presented as means ± standard deviation (SD) of triplicates from 3-6 independent experiments. Statistical analysis of data with Graphpad Prism software version 8.0 was performed using the one-way analysis of variance (ANOVA) followed by Dunnett's post hoc test for multiple comparisons. A *p* value less than 0.05 was considered to be statistically signiﬁcant.

## 3. Results

### 3.1. Cytotoxicity of AVME

The cytotoxicity of AVME was tested in five tumoral (MCF-7, MDA-MB-231, 4T1, SK-MEL-28, and SF-295) and three nontumoral cell lines (NIH-3T3, HUVEC, and MRC-5) (Table 2). After 24 h of exposure, AVME registered a significant cytotoxicity on different tumoral cell lines with a maximum pronounced effect against 4T1 (CC_50_ = 18 ± 1.51 *µ*M) and SK-MEL-28 (CC_50_ = 18 ± 08 *µ*M) cells, followed by MDA-MB-231 (CC_50_ = 20 ± 1.12 *µ*M), MCF-7 (CC_50_ = 21 ± 2.5 *µ*M), and SF-295 (CC_50_ = 21 ± 1.03 *µ*M) cells. Concerning nontumoral cell lines, except murine ﬁbroblast cells (NIH-3T3) where the CC_50_ was also pronounced (21 ± 0.89 *µ*M), the cytotoxicity was of lesser magnitude in other two nontumoral cell lines [HUVEC (CC_50_ = 27 ± 1.27 *µ*M) and MRC-5 (CC_50_ = 30 ± 4.28 *µ*M)]. The selective cytotoxicity index (SCI) was around 1.3 showing greater selectivity of AVME to cancer cells.

### 3.2. Cell Death Induced by AVME

**Figure 1:**
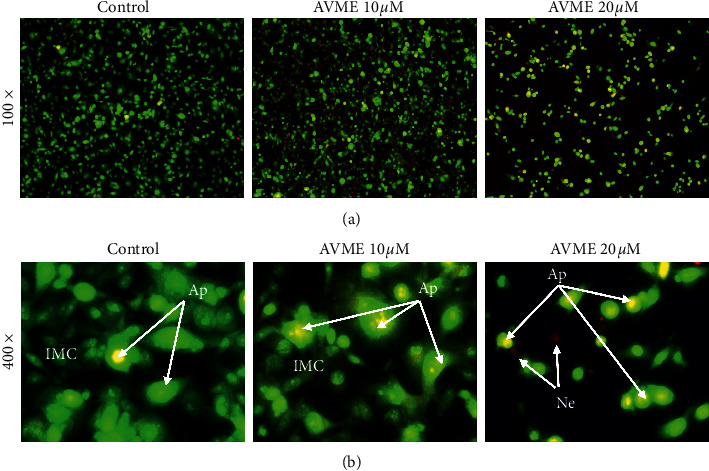
Effect of AVME on cell death in MDA-MB-231 cells (qualitative evaluation). Representative fluorescence microscopic images 100× (a) and 400× cells (b) double-stained with acridine orange and ethidium bromide. IMC: intact membrane cell, Ap: apoptotic cells, Ne: necrotic cells.

Figures 1(a) and 1(b) show MDA-MB-231 cells stained with AO and EB.  Figure 1(b) illustrates condensed green nuclei (apoptotic cell), indicating chromatin condensation. Further, the cell death mechanisms induced by AVME were evaluated through flow cytometry.

**Figure 2:**
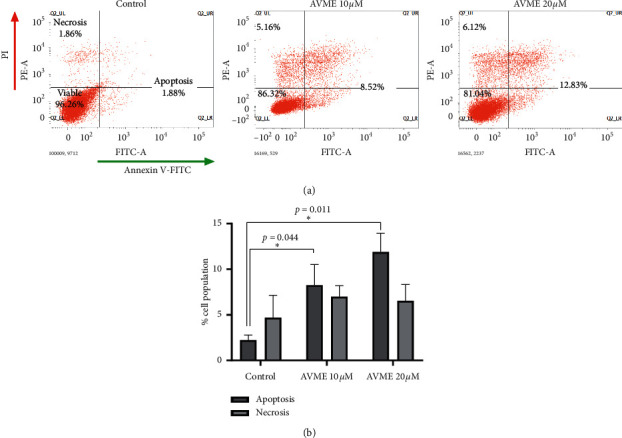
Effect of AVME on cell death in MDA-MB-231 cells (quantitative evaluation). Dot plot (a) representative of one experiment of apoptosis measurement by Annexin-V-FITC/PI staining MDA-MB-231 cells. Cells were treated for 24 h with AVME at concentrations of 10 and 20 *µ*M. The graph (b) shows the percentage of cells in each phase of 3 independent experiments. ^*∗*^*p* < 0.05.

As shown in dot plots (Figure 2(a) and Supplementary Figure 1(a)), the viable cells exhibited low FITC and propidium iodide (PI) fluorescence, whereas the cells in early apoptosis presented high FITC fluorescence but low PI fluorescence. The viable, apoptotic, and necrotic control cells were 96.26%, 1.88%, and 1.86%, respectively. The concentration-dependent increase in the percentage of apoptotic cells with the maximum of 12.83% at 20 *µ*M of AVME suggests that it induced apoptosis in MDA-MB-231 cells (Figure 2(b)) and 23.13% (at 11 *µ*M) and 53.24% (at 21 *µ*M) of apoptotic cells in MCF-7 cells (Supplementary Figures 1(a) and 1(b)).

### 3.3. Cell Cycle

**Figure 3:**
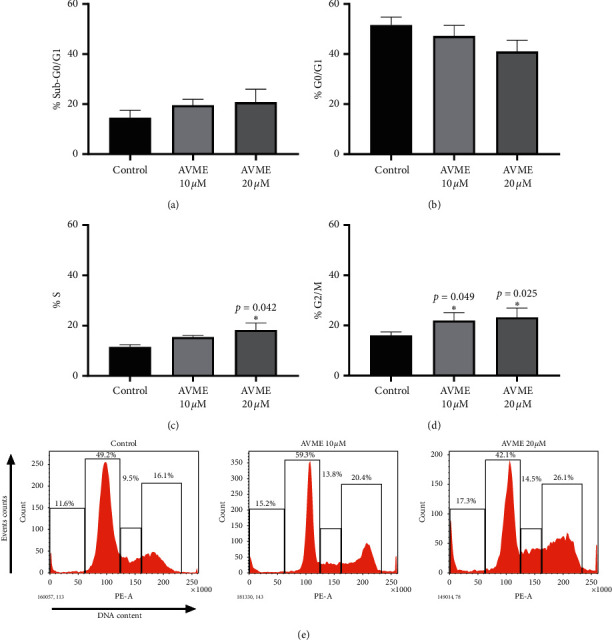
Effect of AVME on cell cycle distribution in MDA-MB-231 cells after 24 h. Cells were treated with 10 and 20 *µ*M of AVME for 24 h and stained with PI. Following flow cytometry, cellular DNA profile was analyzed using the software WinMDI 2.9. (a–d) Results are expressed as the percentage of cell in each cell cycle phase of three independent experiments. ^*∗*^*p* < 0.05. (e) Data representing the percentage of cell counts in each cell cycle phase are also shown.

The effects of AVME on cell cycle progression of MCF-7 and MDA-MB-231 cells were assessed by flow cytometric analysis. AVME significantly increased the percentage of cell population at G2/M and S phases in MDA-MB-231 cells in a concentration-dependent manner (Figures 3(c) and 3(d)).

AVME did not induce significant change in MCF-7 cell cycle after 24 h of incubation at the concentrations of 5, 11, and 21 *µ*M (Supplementary Figure 2).

### 3.4. Mitochondrial Transmembrane Potential

**Figure 4:**
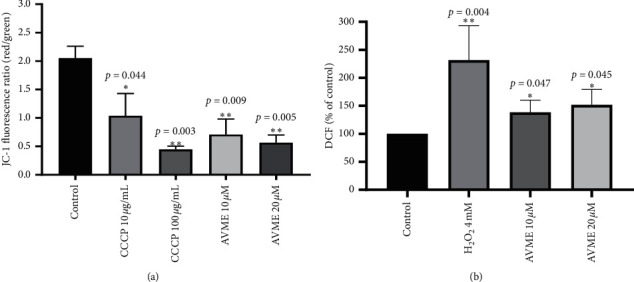
Effects of AVME on mitochondrial transmembrane potential (a) and intracellular ROS levels (b) in MDA-MB-231 cells treated with 10 and 20 *µ*M of AVME. The mitochondrial membrane potential was spectrophotometrically measured using the JC-1 fluorescent probe, while ROS level was determined using the DCHF-DA fluorescent probe. The uncoupler CCCP (10 and 100 *µ*g/mL) and H_2_O_2_ (4 mM) were used as positive control for the mitochondrial transmembrane potential and the ROS determination, respectively. Results are expressed as mean ± SD of three independent experiments. ^*∗*^*p* < 0.05 and ^*∗∗*^*p* < 0.01 as compared to control.

Since AVME was capable of inducing apoptosis, we decided to examine whether it acts via reactive oxygen species- (ROS-) mediated mitochondrial dysfunction pathway. The JC-1 probe emits fluorescence peaks with strong (red) or low (green) mitochondrial membrane potentials. AVME significantly (*p*=0.009 and *p*=0.005) decreased mitochondrial transmembrane potential of MDA-MB-231 cells at the tested concentrations (10 and 20 *µ*M), as evidenced by a decrease of red/green fluorescence ratio. Similar effects were achieved with 5,5′,6′,6-tetrachloro-1,1′,3,3′-tetraethylbenzymidazolcarbocianyne iodide (CCCP) (10 and 100 *µ*M) used as positive control in this experiment (Figure 4(a)).

### 3.5. ROS Levels

Changes in intracellular ROS levels were detected using a fluorescence probe, 2′,7′-dichlorofluorescein diacetate (DCFH-DA). DCFH is normally produced via hydrolysis by intracellular esterases followed by its oxidation to DCF. The fluorescence intensity resembles the level of ROS. Significant increases in ROS levels in MDA-MB-231 cells were observed after incubation with AVME at concentrations of 10 *µ*M (*p*=0.047) and 20 *µ*M (*p*=0.045), and similar effects were found in the positive control assay where the cells were exposed to H_2_O_2_ (4 mM) (Figure 4(b)).

### 3.6. Caspases Activity

**Figure 5:**
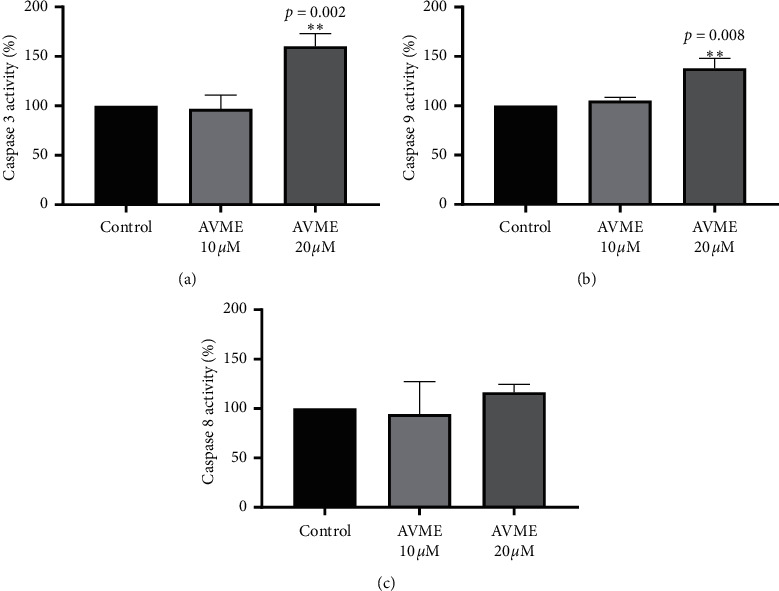
Activation of caspase-3 (a), caspase-9 (b), and caspase-8 (c) by AVME in MBA-MD-231 cells. Cells were incubated with 10 and 20 *µ*M AVME for 8 h. Caspase activities were measured by monitoring the cleavage of fluorogenic substrates specific for each caspase. The activity is given as percentage. Results are expressed as the mean ± SD of three independent experiments. ^*∗∗*^*p* < 0.01 as compared to control.

In further investigation of how AVME activates apoptosis in MDA-MB-231 cells, the activities of caspase-3, -8, and -9 were determined. A significant increase in the activities of caspase-3 (50%) and caspase-9 (45%) was observed after 8 h of cell incubation with AVME at the concentration of 20 *μ*M, whereas it failed in caspase-8 activity (Figure 5).

### 3.7. Bcl-2 Family Protein Expression

**Figure 6:**
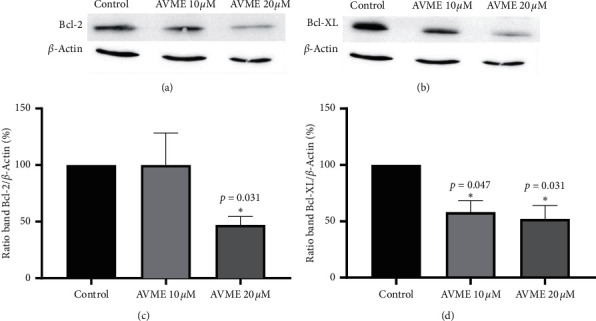
Effects of AVME on Western blot analysis of Bcl-2 (a, c) and Bcl-XL (b, d) protein contents in MDA-MB-231 cells treated with 10 and 20 *µ*M of AVME for 24 h. For Western blotting, *β*-actin was used as an internal control. (c, d) Results are expressed as mean ± SD of three independent experiments. ^*∗∗*^*p* < 0.05 as compared to control.

Bcl-2 and Bcl-XL proteins are known to be overexpressed in various human cancers and act as suppressors of apoptosis, resulting in the survival of malignant cells. Western blot assay was used to quantify Bcl-2 and Bcl-XL proteins in MDA-MB-231 cells. As illustrated in Figure 6(a), AVME induced a reduction in Bcl-2 and Bcl-XL protein contents in MDA-MB-231 cells in a concentration-dependent manner.

### 3.8. Cell Invasion

**Figure 7:**
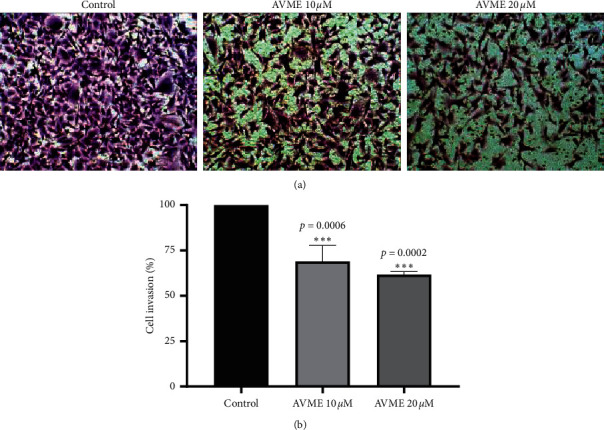
Effect of AVME on invasion of MDA-MB-231 cells. Photomicrographs (100×) of violet crystal staining of MDA-MB-231 cells in transwell insert following 48 h of treatment (a) and results of three independent assays (b). The results are expressed as mean ± SD of three independent experiments. ^*∗∗∗*^*p* < 0.001 as compared to control.

A significant inhibition of invasion of MDA-MB-231 cells was demonstrated by AVME at 10 *µ*M (*p*=0.0006) and 20 *µ*M (*p*=0.0002) in a concentration-dependent manner in comparison with control cells (Figure 7). A 40% reduction of cell invasion was achieved by the maximum concentration of AVME (20 *µ*M) compared to control cells.

### 3.9. Matrix Metalloproteinase Inhibition

**Figure 8:**
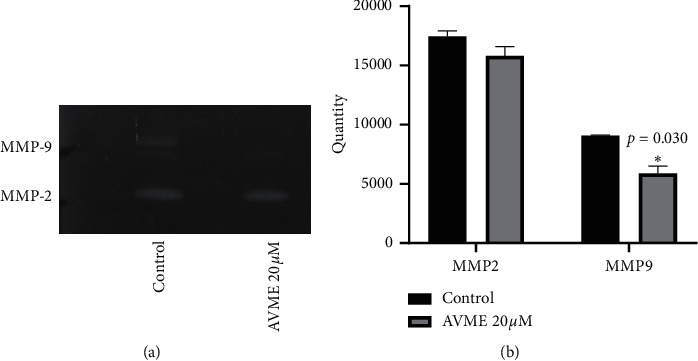
Effects of AVME on zymography assay of MMP-9 and MMP-2 (a) and graph of two independent assays (b) for metalloproteinase matrix activities in MDA-MB-231 cells treated with 20 *µ*M of AVME for 24 h. The results are expressed as mean ± SD of three independent experiments. ^*∗*^*p* < 0.05 as compared to control.

Results of metalloproteinase matrix inhibition obtained from zymography assay are depicted in Figure 8. AVME showed a significant (*p*=0.030) decrease of MMP-9 activity in MDA-MB-231 cells at a concentration of 20 *µ*M but had no significant effect on MMP-2.

## 4. Discussion

Although there is significant progress in the discovery and development of safer antitumoral agents, breast-cancer-related deaths remain high [29]. The identification of new antitumoral phytochemicals led to the increased interest in medicinal plants research [30]. Botanical extracts and their phytoconstituents are known to possess potent oncosuppressive and antitumoral activities. This has been demonstrated through extensive *in vitro* and *in vivo* investigations, especially for plants from arid and semiarid habitats [31–33]. Aimed at contributing to research and development of safer antitumoral agents, the present study explored possible cytotoxicity and associated cellular mechanisms of AVME, a flavanone isolated from the African medicinal plant *Erythrina droogmansiana*.

Several cell-based assays were used to determine the effects of natural substances on cell proliferation and also to demonstrate precise cytotoxic activities that may ultimately result in cell death [34]. Breast cancer is a hormone-related malignancy with remarkable heterogeneity [35]. MCF-7 represents “luminal” type breast cancer cells that express estrogen receptor (ER) and progesterone receptor (PR), whereas MDA-MB-231 is “basal” type and triple-negative (ER-, PR-, and HER2-negative) breast cancer cell line. In this study, AVME exhibited cytotoxic activities for MCF-7 and MDA-MB-231 cells with similar CC_50_ value for both cell lines, suggesting that the cytotoxic effect may be mediated through ER-dependent as well as ER-independent mechanisms. Interestingly, AVME is known to possess a weak phytoestrogenic property [15, 22]. Moreover, burttinone, a structural analogue (with an OH group in the first prenylation) of AVME, has been reported to induce cytotoxic effects against a panel of 60 cancer cell lines [36]. The CC_50_ for burttinone was less than 50 *µ*M against 43 cell lines. Burttinone showed maximum cytotoxicity against colon cancer cell line HCC-2998 (CC_50_ = 20 *µ*M), while the CC_50_ was higher than 50 µM in all the five tested leukemia cell lines. This compound was not selective for any cell of the panel, indicating that it is a general cytotoxic agent with no clinical value [37]. AVME was found to be slightly more cytotoxic against cancerous cells than noncancerous cells, indicating a weak selective activity for cancer cells. In spite of this fact, we believe it is worthwhile to elucidate the underlying mechanisms considering the limitations of current chemotherapeutic drugs regarding undesirable toxicity and resistance development. In this study, it was found that AVME induced apoptosis in MDA-MB-231 and MCF-7 cells (supplementary data). Apoptosis represents one of the targets of the search for alternative treatment against cancer [38]. Generally, apoptosis triggered by either extrinsic or intrinsic mechanisms is known to play a vital role in carcinogenesis and hence can be exploited to identify novel compounds for cancer therapy [39, 40].

Caspase-3 and caspase-9 enzymes, members of cysteine protease family, are indispensable for the breakup of the nuclear scaffold and cytoskeleton [41]. These enzymes play central roles in the process of apoptosis and act as initiators and executioners. The intrinsic mitochondrial pathway can be stimulated by irreparable genetic damage, considerable oxidative stress, and hypoxia, resulting in mitochondrial permeability followed by a release of cytochrome *c* into the cytosol [42]. A balance between proapoptotic proteins (such as Bax, Bad, Bak, and Bcl-Xs) and antiapoptotic proteins (like Bcl-2, Bcl-XL, and Mcl-1) regulates the cytochrome *c* release [42]. The apoptosome complex made up of cytochrome *c*, caspase-9, and Apaf-1 then activates caspase-3 [43]. The increase in caspase-3 and caspase-9 activities following exposure to AVME indicates that this natural compound induces apoptosis by the intrinsic pathway. AVME failed to activate caspase-8, which is activated by a ligand bound to death receptors on the cell surface. The death-inducing signaling complex is known to activate procaspase-8 and caspase-8, constituting the extrinsic pathway of apoptosis [44].

ROS are the products of normal metabolic activities, which are kept fairly constant by regulating their production and elimination. ROS are essential to drive various regulatory pathways [45]. There is mounting evidence that ROS play a pivotal role in the induction of apoptosis via intrinsic mitochondrial cell death pathway or extrinsic death receptor pathway [46]. The ability of high concentrations of ROS to promote mitochondrial membrane pore formation and release apoptogenic molecules is being increasingly exploited by chemotherapeutic agents to induce apoptosis [47, 48]. In this study, ROS levels were increased after incubation of MDA-MB-231 cells with AVME. This result corroborates the findings of Brodská and Holoubek [49] who showed that a significant increase in apoptosis results from an increase in ROS. AVME exhibited a decrease in the transmembrane potential in MDA-MB-231 cells. This finding is in agreement with other studies, which also showed compounds inducing disturbances in mitochondrial membrane potential [50, 51]. After incubation of MDA-MB-231 cells with AVME, a concentration-dependent reduction in the expression of Bcl-XL and Bcl-2 was observed. The reduction in the Bcl-2 proteins content indicates the intrinsic pathway-mediated induction of apoptosis by AVME. Flavonoids are well known proapoptotic compounds, which exert cytotoxicity by the generation of ROS, disruption of mitochondrial transmembrane potential, cytosolic release of cytochrome *c*, decrease in Bcl-2, and ultimate apoptosis [52].

Although CC_50_ values of AVME were ∼20 *µ*M for the breast cancer cells tested, they induced a slight increase of apoptosis cell population (7.9%), suggesting that apoptosis plays a partial role in the AVME-induced cytotoxicity. This has encouraged authors to look for alternative mechanisms. The *in vitro* antitumor effect of AVME was strengthened by the fact that it induced the arrest of MDA-MB-231 cells in G2/M and S phases. The ability to block the cell cycle in the G2/M phase has been exhibited by several phytochemicals with antiproliferative and cytotoxic effects [53]. An important observation of the present study is that AVME-mediated decreased cell migration and invasion. Cell migration is an important step for cancer progression and metastasis [54]. The levels of various proteinases, such as MMP-2 and MMP-9, are known to be increased during inflammatory diseases and in cancer [53]. These proteins are able to damage the extracellular matrix, altering the cell-cell and cell-extracellular matrix interaction in cancer and consequently inducing tumor promotion, invasion, and metastasis [53]. Mehner et al. [55] demonstrated that the MMP-9 knockdown in MDA-MB-231 cells was able to reduce cell invasion in Matrigel transwell assay by 90% while MMP-2 knockdown reduced cell invasion by approximately 50%. Research indicated that MMP-9 is closely related to the invasion of MDA-MB 231 cells. Based on this information, we suggest that AVME may be inhibiting the invasion of MDA-MB-231 cells possibly by suppressing the MMP-9 activity. The precise mechanisms of action of AVME and target proteins (if any) have not yet been discovered, thus giving room for further investigation.

## 5. Conclusions

In summary, our study is the first demonstration of the fact that AVME, a prenylated flavanone isolated from the African medicinal plant *E*. *droogmansiana*, induces cytotoxic effect in breast cancer cell lines by triggering apoptosis via ROS-mediated mitochondrial pathway characterized by activation of caspase-3 and caspase-9 and downregulation of Bcl-2 and Bcl-XL proteins. Moreover, AVME exhibits anti-invasion activities by the inhibition of MMP-9 activity. Nevertheless, further investigations are needed to comprehend the full potential of these results.

## Figures and Tables

**Table 1:** Abyssinone V-4′ methyl ether (AVME) isolated from *Erythrina droogmansiana*.

Chemical names	Crystal color	Structure, molecular weight, and formula
Abyssinone V-4′ methyl ether, 4H-1-benzopyran-4-one, 2,3-dihydro-5,7-dihydroxy-2-[4-methoxy-3,5-bis(3-methyl-s2-buten-1-yl)phenyl]	White	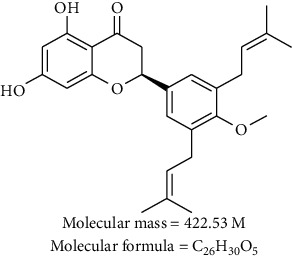

This compound was reported by Tueche et al. [23].

**Table 2:** Comparative CC_50_ and selective cytotoxicity index (SCI) values of AVME in tumoral and non‐tumoral cell lines.

CC_50_ (*µ*M) [mean ± SD]				SCI
Tumoral cell lines		Non‐tumoral cell lines		CC_50_ HUVEC cells/CC_50_ tumoral cells
MCF-7	21 ± 3.51	HUVEC	27 ± 2.51	1.3
MDA-MB-231	20 ± 2.18	MRC-5	30 ± 0.78	1.4
4T1	18 ± 1.11	NIH-3T3	21 ± 0.89	1.5
SK-MEL-28	18 ± 3.1			1.5
SF-295	21 ± 2.03			1.3

CC_50_: concentration of AVME which results in 50% of cell viability. SCI: selective cytotoxicity index. The results are expressed as mean ± SD of at least 3 independent experiments. The results are expressed as mean ± SD of at least 3 independent experiments. Selectivity index: ∼1.3 (CC_50_ nontumoral cells/CC_50_ tumoral cells).

## Data Availability

The data and materials used in this study are available from the corresponding author upon request.

## Abbreviations

AVME: Abyssinone V-4' methyl ether
AO: Acridine orange
BSA: Bovine serum albumin
CC_50_: Cytotoxic concentration which kills 50% of cells
DCFH-DA: 2′,7′-Dichlorofluorescein diacetate
DMBA: 7,12-Dimethylbenz(a)anthracene
DMEM: Dulbecco's modified Eagle medium
EB: Ethidium bromide
ER: Estrogen receptor
IMC: Intact membrane cells
PI: Propidium iodide
PR: Progesterone receptor
GPS: Global positioning system
NHC: National Herbarium of Cameroon
SEM: Standard error of mean.

## Supplementary Materials

Supplementary Materials Supplementary Figure 1: Dot plot (A) representative of one experiment of apoptosis measurement by Annexin-V-FITC/PI staining MCF-7 cells. Cells were treated for 24 h with AVME at concentrations of 11, 21, and 42 *μ*M. The graph (B) shows the percentage of cells in apoptosis or necrosis of 3 independent experiments. Supplementary Figure 2: Effect of AVME on cell cycle distribution in MCF-7 cells after 24 h. Cells were treated for 24 h with 5, 11, and 21 *μ*M of AVME and stained with PI. Following flow cytometry, cellular DNA profile was analyzed using the software WinMDI 2.9. Data represent the percentage of cell counts in sub-G0/G1 (A), G0/G1 (B), S (C), and G2/M (D) phases. Histograms (E) represent one experiment of cell cycle. The results are expressed as the percentage of cells in each cell cycle phase of three independent experiments.
