# Supplementary material for: Abyssinone V-4′ Methyl Ether, a Flavanone Isolated from Erythrina droogmansiana, Exhibits Cytotoxic Effects on Human Breast Cancer Cells by Induction of Apoptosis and Suppression of Invasion
Source: Evid Based Complement Alternat Med. 2020 Jul 23;2020:6454853. doi: 10.1155/2020/6454853 (PMC7396086; doi:10.1155/2020/6454853)
Supplement: Supplementary Materials — Supplementary Figure 1: Dot plot (A) representative of one experiment of apoptosis measurement by Annexin-V-FITC/PI staining MCF-7 cells. Cells were treated for 24 h with AVME at concentrations of 11, 21, and 42 μM. The graph (B) shows the percentage of cells in apoptosis or necrosis of 3 independent experiments. Supplementary Figure 2: Effect of AVME on cell cycle distribution in MCF-7 cells after 24 h. Cells were treated for 24 h with 5, 11, and 21 μM of AVME and stained with PI. Following flow cytometry, cellular DNA profile was analyzed using the software WinMDI 2.9. Data represent the percentage of cell counts in sub-G0/G1 (A), G0/G1 (B), S (C), and G2/M (D) phases. Histograms (E) represent one experiment of cell cycle. The results are expressed as the percentage of cells in each cell cycle phase of three independent experiments. [file 6454853.f1.pdf]

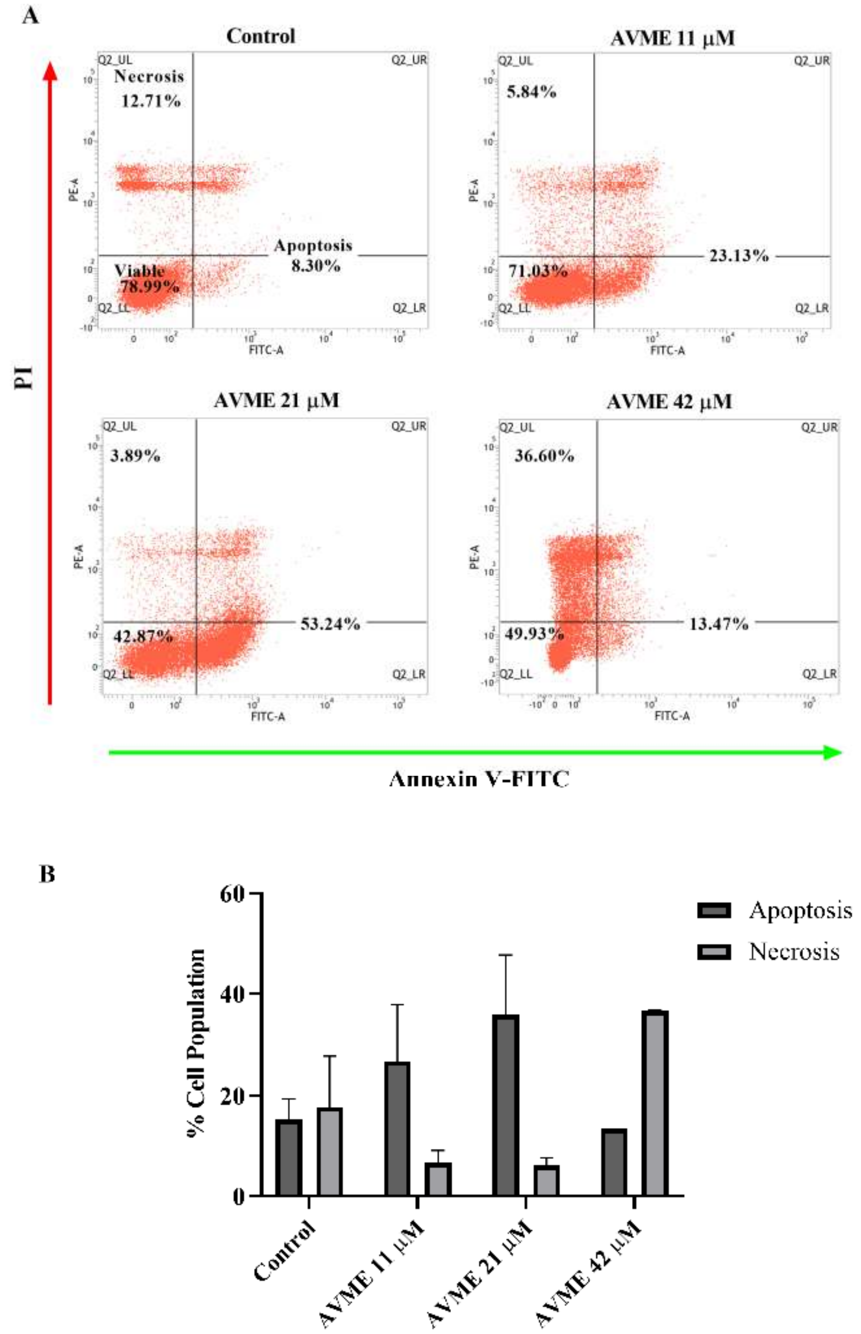

Suppl Figure 1. Dot plot (**A**) representative of one experiment of apoptosis measurement by Annexin-V-FITC/PI staining MCF-7 cells. Cells were treated for 24 h to AVME at concentrations of 11, 21 and 42 μM. The graph (**B**) shows the percentage of cells in apoptosis or necrosis of 3 independent experiments.

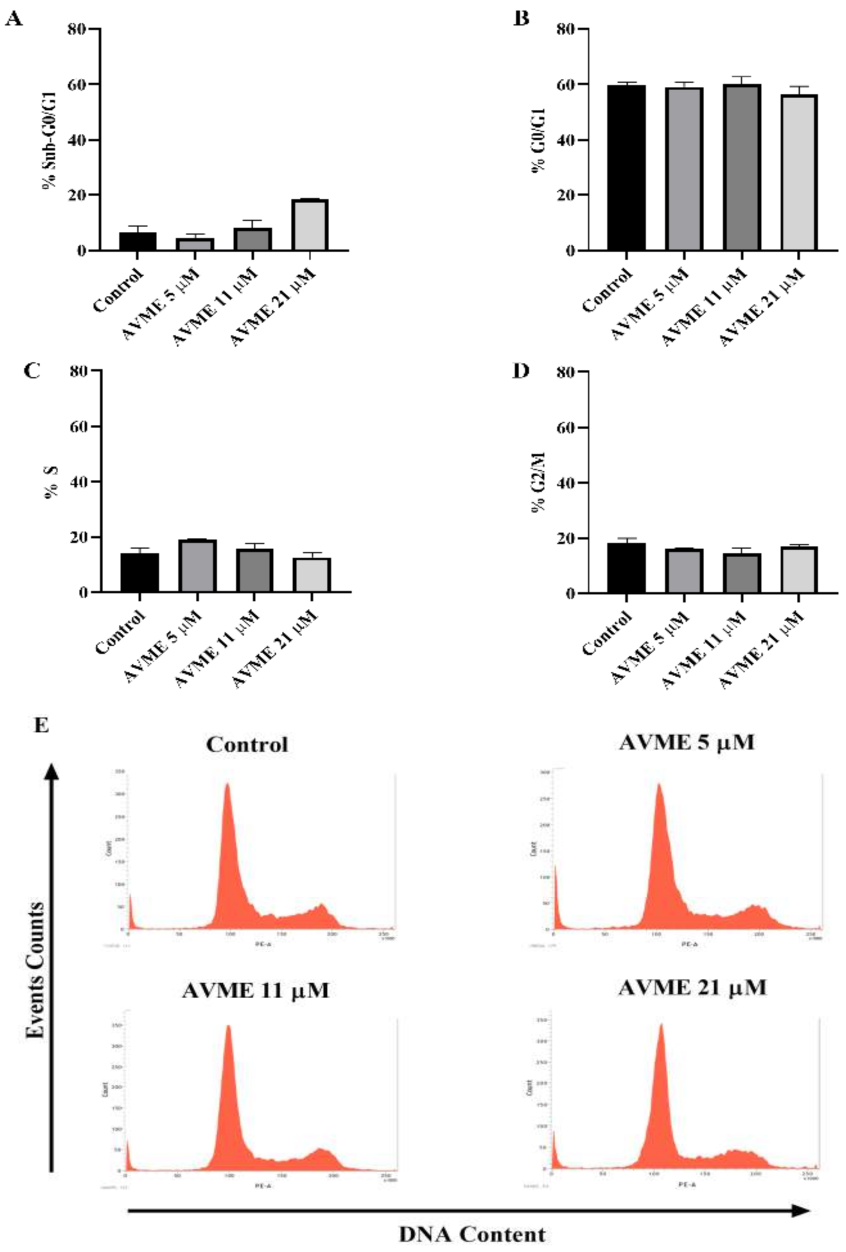

Suppl Figure 2. Effect of AVME on cell cycle distribution in MCF-7 cells after 24 h. Cells were treated for 24 h with 5, 11 and 21 µM of AVME and stained with PI. Following flow cytometry, cellular DNA profile was analyzed using the software WinMDI 2.9. Data representing the percentage of cell counts in Sub-G0/G1 (A), G0/G1 (B), S (C) and G2/M (D) phases. Histograms (E) representative of one experiment of cell cycle. The results are expressed as the percentage of cell in each cell cycle phase of three independent experiments.
